# Dysregulation of macrophage polarization is associated with the metastatic process in osteosarcoma

**DOI:** 10.18632/oncotarget.13055

**Published:** 2016-11-13

**Authors:** Clotilde Dumars, Jean-Michel Ngyuen, Aurélie Gaultier, Rachel Lanel, Nadège Corradini, François Gouin, Dominique Heymann, Marie-Françoise Heymann

**Affiliations:** ^1^ INSERM, UMR 957, Equipe LIGUE Nationale Contre le Cancer, Nantes, France; ^2^ Université de Nantes, Nantes atlantique universités, Pathophysiology of Bone Resorption and Therapy of Primary Bone Tumors, Nantes, France; ^3^ CHU de Nantes, Nantes University Hospital, France; ^4^ Centre de Lutte Contre le Cancer, Léon Bérard, Lyon, France; ^5^ INSERM, European Associated Laboratory “Sarcoma Research Unit”, Department of Oncology and Metabolism, University of Sheffield, Medical School, Sheffield, UK

**Keywords:** osteosarcoma, tumour associated macrophage, osteoprotegerin, Pathology Section

## Abstract

Osteosarcoma (OS) is the most common bone sarcoma in adolescents, and has poor prognosis. A vicious cycle is established between OS cells and their microenvironment in order to facilitate the tumor growth and cell spreading. The present work aims to better characterize the tumor microenvironment in OS in order to identify new therapeutic targets relating to metastatic process. Tissue microarrays of pre-chemotherapy OS biopsies were used for characterizing the tumor niche by immunohistochemistry. Parameters studies included: immune cells (M1, M2-subtypes of tumor-associated macrophages (TAM); T, B lymphocytes; mast cells), vascularization (endothelial, perivascular cells), OPG, RANKL, and mitotic index. Two groups of patients were defined, 22 localized OS (OS Meta-) and 28 metastatic OS (OS Meta+). The OS Meta- group was characterized by a higher infiltration of INOS^+^ M1-polarizedmacrophages and upregulated OPG immunostaining. OS Meta+ tumors showed a significant increase in CD146^+^ cells. INOS^+^ M1-macrophages were correlated with OPG staining, and negatively with the presence of metastases. CD163^+^ M2-macrophages were positively correlated with CD146^+^ cells. In multivariate analysis, INOS and OPG were predictive factors for metastasis. An older age, non-metastatic tumor, good response to chemotherapy, and higher macrophage infiltration were significantly associated with better overall survival. TAMs are associated with better overall survival and a dysregulation of M1/M2 polarized-macrophages in favor of M1 subtype was observed in non-metastatic OS.

## INTRODUCTION

Primary bone sarcomas are rare oncologic subtypes, accounting for less than 0.2% of the malignant tumors registered in the EUROCARE database [1]. These tumors are made up of a large number of distinct histological entities, especially osteosarcoma (OS), chondrosarcoma and Ewing sarcoma originating from mesenchymal stem cells [2, 3]. OS is the most common bone sarcoma in young patients with a peak of incidence at 18 years. Secondary OS occurs in the elderly mainly after Paget disease or radiotherapy. The standard treatment involves both surgery and chemotherapy, but is unfortunately ineffective in many cases, due to the development of drug refractory and/or resistance cells, leading to the development of metastasis and death. Radiotherapy may be used in palliation [1]. Consequently, OS still has a poor prognosis and patient survival is strongly associated with the tumor cell response to chemotherapy, and metastatic status. A 5-year survival rate of 70% is observed for patients with non-metastatic OS, and 30% for patients with metastatic OS at diagnosis are still alive at the end of the five-year period [4]. Understanding the pathophysiology of OS and the metastatic process is a pre-requisite for future improvement in therapeutic approaches.

The pathogenesis of OS is closely related to the microenvironment in which the tumor grows. Even if the etiology of OS has not been clearly established, its development has the special feature of being strongly associated with its microenvironment and, more specifically, with the bone niche. There is effectively dysregulation in the balance between Osteoprotegerin (OPG) / Receptor Activator of NF-κB (RANK) / RANK Ligand (RANKL), provoking exacerbated local bone remodeling. As a result, numerous factors initially trapped in this matrix are released, which in turn stimulate sarcoma cell proliferation, leading to the establishment of a vicious cycle between bone and tumor cells [5]. These events are associated with early and late events in the metastatic process by promoting the neoangiogenesis and extravasation of tumor cells [6, 7]. The sarcoma tumor niche, as with hematologic disorders, is also considered to be a sanctuary for tumor cell expansion, and drug resistance leading to cell dissemination [8–10].

The immune niche, with its huge cell diversity including more specifically tumor-infiltrating lymphocytes and tumor-associated macrophages (TAMs), regulates the OS microenvironment [11, 12]. TAMs exert different effects on tumor development because of their polarization. In oncology, M1-polarized macrophages are considered to be anti-tumor effectors and M2-polarized macrophages are defined as pro-tumor modulators as they increase the neoangiogenic process [13–15]. The density of TAMs is correlated with tumor cell proliferation, invasion, metastasis, and poor prognosis in various epithelial and hematological cancers, and in bone metastases [16].

The tumour microenvironment is suspected to play a regulatory function of OS cells and could be a potential source of therapeutic targets, unfortunately its characterization has not been very well documented [17, 18]. In this context, the aim of the present work was to characterize the tumor “niche” of localized and metastatic human OS by means of histopathological assessment in a large biological cohort associated with clinical annotations. The final goal was to identify new prognostic information in OS, and to specify therapeutic targets.

## RESULTS

### Patient characteristics and tumor features

Between 1994 and 2013, 159 patients treated for OS were identified in the database of the Nantes University Hospital (123 patients without metastasis, and 36 with metastatic disease). Twenty-two patients without available pre-chemotherapy samples were excluded (16 OS Meta- and 6 OS Meta+) and 13 patients whose diagnosis changed following surgical resection of the specimen or reviewing the slides (11 OS Meta-, 2 OS Meta+). For the OS Meta- group, 40 patients diagnosed after 2008, 11 patients with recurrence, 17 with metastatic evolution with no available samples, and 6 without follow-up were excluded. At the end of the selective process, 22 and 28 patients were finally included in the OS Meta- and OS Meta+ populations respectively. The demographic, clinical and histological data of these 50 patients are shown in Table 1. The median age at diagnosis was similar between the two groups (22.5 for OS Meta-, 23.7 for OS Meta+). Tumor location (metaphysis of a long bone) and the distribution of histological subtypes (conventional OS including osteoblastic, chondroblastic and fibroblastic OS) were also similar between the two groups. The number of male and female patients was similar in the OS Meta- group, and males were predominant in the OS Meta+ group (19/9). Metastatic lesions were metachronous in 72% of the cases and the main metastatic site was the lung (75%). The survival rate was significantly higher in the OS Meta- group (p = 0.0003).

**Table 1:** Characteristics of osteosarcoma patients included in the study

	OS Meta- (*n*=22)	OS Meta+ (*n*=28)	*p*
**Age**, mean (years), (min-max)	22.52 (7-76)	23.70 (8-80)	0.7616
Sexe, *n* (%) Female Male	11 (50) 11 (50)	9 (32) 19 (68)	0.2514
**Primary tumor site**, *n* (%) Femur Tibia/fibulae Humerus Ulna Others	11 (50) 5 (22,5) 5 (22,5) 1 (5) 0	17 (61) 6 (21) 3 (11) 0 2 (7)	0.6292
Size, mean (cm), (min-max)	9.51 (3-26.5)	11.2 (4.8-31)	0.2143
**Histological subtype**, *n* (%) Fibroblastic Osteoblastic Chondroblastic Composite Telangiectatic Parosteal Secondary	5 (20) 11 (50) 2 (10) 0 1 (5) 2 (10) 1 (5)	7 (25) 13 (46) 3 (10.5) 3 (10.5) 1 (4) 0 1 (4)	0.6887
**Neo-adjuvant chemotherapy**, *n* (%)	19 (86)	26 (93)	0.3849
**Histological response to chemotherapy**, *n* (%) I II III	4 (22) 5 (28) 9 (50)	8 (31) 8 (31) 10 (38)	0.7381
**Soft tissue invasion**, *n* (%)	17 (77)	26 (93)	0.2171
**Quality of resection**, *n* (%) R0 R1	20 (91) 2 (9)	22 (79) 6 (21)	0.4391
**Metastases**, *n* (%) Synchronous Metachronous	-	8 (28) 20 (72)	
**Metastatic location**, *n* (%) Lung Bone Lymph Node Multiple	-	21 (75) 3 (10.5) 1 (4) 3 (10.5)	
Size, mean (cm), (min-max)	-	3 (0.4-9)	
**Quality of resection**, *n* (%) R0 R1	-	21 (95.5) 1 (4.5)	
**Death**, *n* (%)	2 (9)	20 (71)	*<0.005*

### The immune infiltrate of OS Meta- is enriched with M1-polarized macrophages compared to OS Meta^+^: relationship with the metastatic process

As immune cells are associated with control of the oncogenic process, we first characterized the immune infiltrate in OS samples selected by immunohistochemistry (Table 2). T and B lymphocyte infiltration were moderately detected in all samples studied and were not significantly different between the two OS groups. T lymphocytes were organized in clusters in contrast to the B lymphocyte population which was scattered throughout tumor tissues (Supplementary Figure 1). Immune infiltrate was also composed of a limited number of mast cells identified by the CD117 antibody, with thin granular cytoplasmic staining (Supplementary Figure 1).

**Table 2:** Immunohistochemical analysis of osteosarcomas

Antigen studied		OS Meta- (*n* = 22)	OS Meta+ (*n* = 28)	*p*
CD3^*^ median (min-max)		2.3 (0-40)	2 (0-14)	0.510
CD20^*^ median (min-max)		0 (0-3.6)	0 (0-2.6)	0.528
CD4^*^ median (min-max)		0 (0-1.6)	0 (0-4)	0.894
CD8^*^ median (min-max)		3 (0-22)	1.83 (0-19.6)	0.188
CD68^*^ median (min-max)		28.5 (1.5-84.6)	19.3 (5.5-71.3)	0.148
INOS^*^ median (min-max)		3 (0-47)	0 (0-8)	***0.001***
CD163^*^ median (min-max)		1.2 (0-55)	0.5 (0-8.3)	0.265
CD117^*^ median (min-max)		1 (0-7.3)	0.6 (0-5)	0.15
Ki-67^**^ median (min-max)		3.2 (0-63.3)	1.7 (0-40)	0.379
CD31^***^ *n* (%)	Density score 0 1 2 3	5 (22.5) 12 (55) 5 (22.5) 0 (0)	7 (25) 7 (25) 11 (39) 3 (11)	0.108
SMA^***^ *n* (%)	Density score 0 1 2 3	1 (5) 17 (77) 4 (18) 0 (0)	1 (3.5) 21(75) 5 (18) 1 (3.5)	1
CD146^***^ *n* (%)	Density score 0 1 2 3	0 (0) 19 (86.4) 3 (13.6) 0 (0)	2 (7.1) 13 (46.5) 11 (39.3) 2 (7.1)	***0.014***
OPG^***^ *n* (%)	Density score 0 1 2 3	1 (4.5) 1 (4.5) 5 (23) 15 (68)	0 (0) 10 (36) 5 (18) 13 (46)	***0.028***
RANKL^***^ *n* (%)	Density score 0 1 2 3	0 (0) 1 (4.5) 6 (27.5) 15 (68)	0 (0) 7 (25) 8 (29) 13 (46)	0.130

OS Meta- : patients with non metastatic osteosarcoma; OS Meta+: patients with metastatic osteosarcoma.

* Number of cytoplasmic stained cells for 1 high power(x40) microscopic field (HPF) estimated on 3 « hot-spots ».

** Number of stained nuclear on one HPF.

*** Semi-quantitatively analysis of the vascular or cellular density (0, no staining; 1, < 1/3 of the surface; 2, between 1/3 and 2/3 of the surface; 3, >2/3 of the tumor surface).

**Figure 1:**
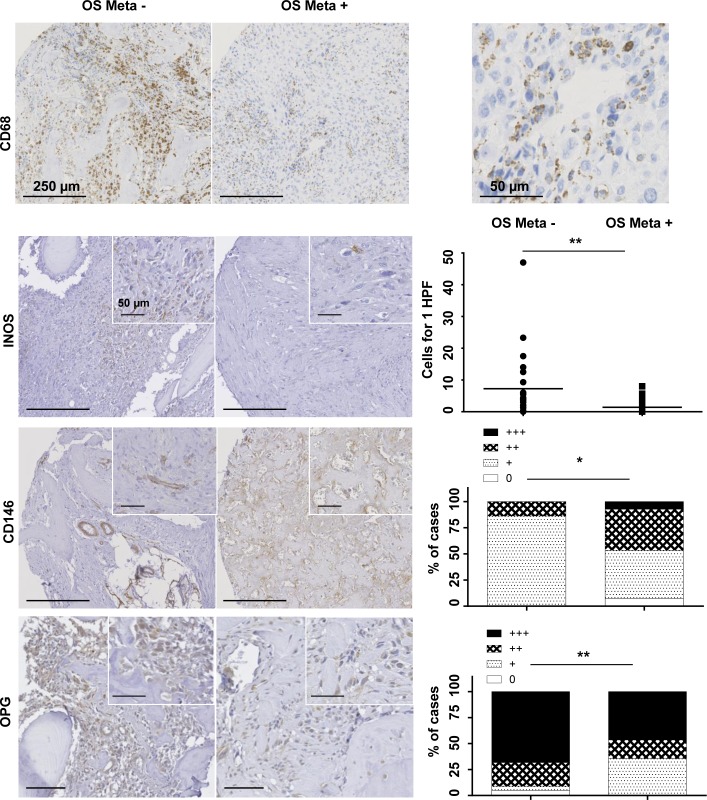
Representative immunohistochemical results of CD68, INOS, CD146 and OPG, in primary lesions of OS Meta- and OS Meta+ The immunohistochemical study was performed on tissue micro-arrays and analyzed on digitized images. Macrophages are the main cells in the bone niche; the qualitatively analysis revealed their perivascular location (Bar scale 250 and 50 µm, original magnification x10 and x40 respectively). Quantitative analysis showed a significantly higher infiltration of M1-polarized macrophages in OS Meta- patients [number of INOS+ cells for 1 high power (x40) microscopic field (HPF) estimated on 3 “hot-spots“; median value symbolized with a horizontal bar] (Fisher test, p = 0.001). On the contrary, OS Meta+ had a significantly higher vascular density (CD146 staining) (Wilcoxon test, p = 0.014) and OPG density was significantly higher in the OS Meta- group (Wilcoxon test, p = 0.028). (Semi-quantitative analysis of vascular or cellular density: 0, no staining; 1, < 1/3 of the surface; 2, between 1/3 and 2/3 of the surface; 3, >2/3 of the tumor surface). OS Meta-: patients with non-metastatic OS; OS Meta+: patients with metastatic OS

TAMs have been shown to control numerous biological processes such as cancer cell growth, neoangiogenesis and activation of T lymphocytes. We thus analyzed TAM infiltration in OS samples (Table 2 and Figure 1). TAMs were frequently located around the blood vessels (Figure 1). While the total number of CD68 macrophages was similar in both OS populations, a differential polarization of these macrophages was observed (Table 2). Interestingly, the number of M1-polarized macrophages (INOS ^+^) was higher in OS Meta- compared to OS Meta+ (p = 0.001) (Table 2 and Figure 1). In addition, in multivariate analyses, INOS was a predictive factor for OS Meta- (p = 0.0298) (Supplementary Table 2).

### Vascular density is higher in OS Meta^+^ compared to OS Meta- in contrast to OPG expression

The metastatic process is strongly associated with neovascularization. Vasculature markers were thus studied in the OS samples (Table 2 and Figure 1). In contrast to CD31 and SMA immunopositivity, which were similar in both OS groups, CD146 was higher in the OS Meta+ population compared to OS Meta- (Figure 1, p = 0.014). This higher vascular density was in favor of an upmodulation of the neoangiogenic process. We then analyzed OPG expression, a key modulator for bone resorption, immune and vascular cells [19]. OPG was overexpressed in the OS Meta- group compared to the OS Meta+ population, and was mainly expressed by OS cells (Figure 1, p = 0.028). In multivariate analyses, OPG was a predictive factor for OS Meta- (p = 0.0367) (Supplementary Table 2).

### Correlation study between the various biomarkers in the OS niche

**Figure 2:**
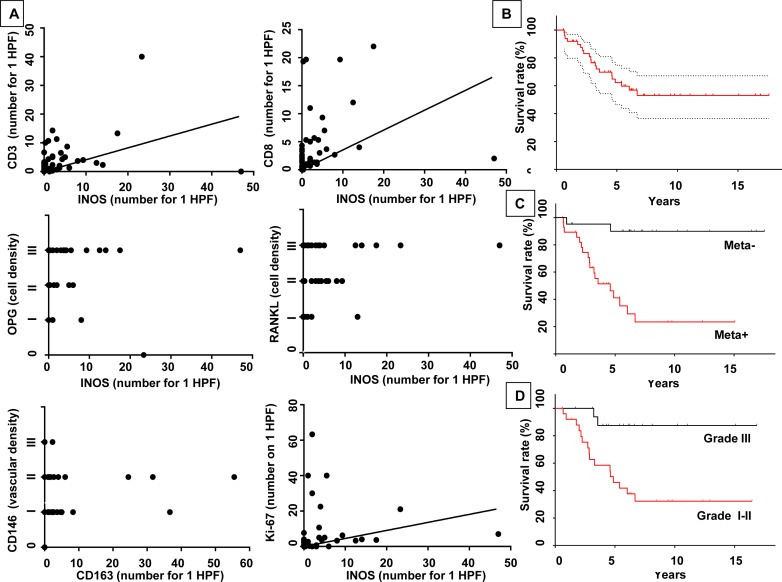
INOS+ M1-polarized macrophages correlate CD3+ CD8+ lymphocytes, OPG, RANKL and the mitotic index and M2-polarized macrophage with the vascularization in primary lesions of OS **A**. A positive correlation was found between INOS^+^ M1-polarized macrophages and CD3^+^CD8^+^ lymphocytes, OPG, RANKL and the mitotic index (Spearman correlation, p = 0.00151, p = 0.03131, p = 0.0150, p = 0.00218 respectively). M2-polarized macrophages as determined by CD163^+^ cells, were correlated with vascularity as determined by CD146^+^ cell density (Spearman correlation, p = 0.00154). Kaplan-Meier Curve of survival, according to presence of metastasis at diagnosis, and of response to chemotherapy: **B**. Median overall survival was 5.12 years (95%CI: 4.62-7.12). **C**. Patients with metastasis (synchronous or metachronous) were associated with significantly lower median overall survival [3.26 years (95%CI: 3.78-7.18)], p < 0.001. **D**. Patients with a worse histological response to chemotherapy [defined as < 90% of tumor necrosis after neoadjuvant chemotherapy] were associated with worse overall survival [4.61 years (95%CI: 3.78-7.18)], p < 0.01. Meta-: non-metastatic patients; Meta+: metastatic patients; Grade III, I-II according the Huvos score.

The correlation analysis between the biological markers in the OS niche revealed various significant relationships (Table 3). Macrophage markers were positively correlated to each other (INOS/CD68 Spearman coefficient SC = 0.599 p < 0.0001, CD68/CD163 SC = 0.587 p < 0.0001, CD163/INOS SC = 0.451 p = 0.00113). In addition, a significant correlation was observed with INOS^+^ M1-macrophages and with CD3^+^ and CD8^+^ T lymphocyte markers (SC = 0.445, p = 0.00151) (Figures 2A), with mast cells (SC = 0.403 p = 0.00407) (Supplementary Figure 1A), with OPG staining (SC = 0.308, p = 0.03131) (Figure 2A) and RANKL (SC = 0.345, p = 0.01503) (Figure 2A). Interestingly, INOS^+^ M1-macrophages were negatively correlated with the presence of metastasis (OR = 0.736, p = 0.01926) (II), while they correlated positively with the Ki-67 mitotic index (SC = 0.428, p = 0.00218) (Figure 2A). CD163^+^ M2-macrophages were correlated with CD146^+^ vascular cells (SC = 0.341, p = 0.01543) (Figure 2A).

**Table 3:** Correlation analysis between the biological markers of osteosarcoma microenvironment

	CD3	CD20	CD4	CD8	CD68	INOS	CD163	CD117	CD31	SMA	CD146	Ki-67	OPG	RANKL
**CD3**		*coeff* *p*	*coeff* *p*	*coeff* *p*	*coeff* *p*	*coeff* *p*	*coeff* *p*	*coeff* *p*	*coeff* *p*	*coeff* *p*	*coeff* *p*	*coeff* *p*	*coeff* *p*	*coeff* *p*
**CD20**	0.488 ***<0.005***	/												
**CD4**	0.396 **0.005**	0.052 0.723	/											
**CD8**	0.521 ***<0.005***	0.377 **0.008**	0.148 0.309	/										
**CD68**	0.545 ***<0.005***	0.432 ***<0.005***	0.211 0.146	0.638 ***<0.005***	/									
**INOS**	0.370 **0.009**	0.217 0.134	0.173 0.240	0.445 ***<0.005***	0.599 ***<0.005***	/								
**CD163**	0.303 **0.034**	0.142 0.326	0.239 0.098	0.357 **0.012**	0.587 ***<0.005***	0.451 ***<0.005***	/							
**CD117**	0.394 **0.005**	0.013 0.926	0.043 0.766	0.427 ***<0.005***	0.216 0.131	0.403 ***<0.005***	0.088 0.543	/						
**CD31**	0.249 0.085	0.048 0.743	0.234 0.105	0.195 0.179	0.159 0.270	0.218 0.132	0.143 0.320	0.109 0.450	/					
**SMA**	0.276 0.055	−0.113 0.434	0.106 0.468	0.247 0.088	0.140 0.334	0.235 0.104	0.039 0.789	0.211 0.142	0.368 0.009*	/				
**CD146**	0.282 **0.049**	0.128 0.377	0.238 0.099	0.248 0.086	0.422 ***<0.005***	0.113 0.437	0.341 **0.015**	−0.056 0.697	0.408 ***<0.005***	0.385 **0.006**	/			
**Ki-67**	0.383 ***<0.005***	−0.004 0.980	0.258 0.074	0.251 0.082	0.305 **0.031**	0.428 ***<0.005***	0.514 ***<0.005***	0.255 0.074	0.324 **0.021**	0.072 0.617	0.096 0.509	/		
**OPG**	0.130 0.372	−0.041 0.777	0.050 0.732	0.066 0.653	0.129 0.373	0.308 **0.031**	0.208 0.147	0.334 **0.018**	0.095 0.512	0.272 0.056	−0.126 0.383	0.096 0.509	/	
**RANKL**	0.166 0.254	−0.087 0.548	−0.006 0.964	−0.085 0.560	0.218 0.128	0.345 **0.015**	0.207 0.149	0.263 0.065	0.166 0.248	0.159 0.270	0.098 0.498	0.098 0.498	0.522 ***<0.005***	/
**Metastasis**	0.937 0.273	0.692 0.314	1.566 0.335	0.919 0.151	0.973 0.088	0.736 **0.019**	0.990 0.135	0.746 0.085	1.627 0.157	1.346 0.599	2.648 0.072	0.973 0.287	0.541 0.087	0.436 0.056

The mitotic index was also correlated with CD3^+^ T lymphocytes (CS = 0.383, p = 0.00664), and CD31^+^ vascular cells (SC = 0.324, p = 0.02181). Each vascular marker was positively correlated (CD31/SMA SC = 0.368 p = 0.00858; SMA/CD146 SC = 0.385 p = 0.00581; CD146/CD31 SC = 0.408 p = 0.00327). CD146^+^ cells were correlated with T lymphocytes CD3^+^ (CS = 0.282, p = 0.04939) (Table 3). Finally, a positive correlation was found between OPG and RANKL (SC = 0.522, p = 0.0001), and between OPG and mast cells (SC = 0.334, p = 0.01765) (Supplementary Figure 1B).

### Clinical and biological/histological factors associated with survival

The median overall survival for all patients was 5.12 years (95% IC: 4.62-7.12) (Figure 2B). Oldest age at diagnosis is significantly associated with better overall survival (p < 0.0001) as well as a non-metastatic status (grade III-IV, Huvos score) [6.90 years (95% IC: 6.06-10.27) vs 3.26 years (95% IC: 2.84-5.31); p = 0.000903 respectively](Figure 2C). As expected, a good response to chemotherapy was also associated with a significant survival rate respectively and [5.72 years (95%IC: 4.46-8.5) vs 4.61 years (95%IC: 3.78-7.18), (p = 0.00702)] (Figure 2D). Among the histological markers in primary tumors, a higher macrophage infiltration tumor was significantly associated with better overall survival (p = 0.04609) (Supplementary Table 3).

## DISCUSSION

In the absence of any clear etiology for OS, the concept of tumor niche has emerged based on the seed and soil theory proposed by Paget at the end of the 19^th^ century [20, 21]. The tumor niche is defined as a specific microenvironment promoting the emergence of cancer-initiating cells, and providing all the factors required for their quiescence, proliferation and migration. A favorable tumor niche associated with one or more oncogenic events may thus explain the pathogenesis of OS. The present work aimed to better characterize the microenvironment of OS by comparing non-metastatic and metastatic OS. Our study demonstrated a differential composition of the tumor niche between the OS Meta- and OS Meta+ groups. A significant increase in M1-polarized macrophage infiltration and OPG immunostaining was observed in OS Meta- compared to OS Meta+. In contrast, OS Meta+ exhibited a significantly higher vascular density compared to the OS Meta- group. Based on these observations, INOS and OPG were identified as predictive factors for non-metastatic disease in multivariate analyses. These results might make possible better understanding of the pathophysiology of OS, and may lead to new therapeutic options (e.g. stimulation of the differentiation of TAM toward M1 macrophages and/or recruitment of M1 macrophages).

In the immune cell population, macrophages are the most numerous in the OS tumor niche, regardless of the metastatic status of the patients. The exact role of macrophages in OS is still unclear and controversial. Some studies have defined TAMs as anti-tumoral effectors. Buddingh et al. thus demonstrated that higher TAM infiltration was associated with better overall survival in high-grade OS [22]. However, the authors did not observe any differences between metastatic and non-metastatic OS, and TAMs exhibited both M1 and M2 characteristics [22]. On the contrary, the impact of macrophages in tumor development has been also suspected. Lewis and Pollard distinguished the anti-tumor M1-macrophages from M2-macrophages leading to tumor growth and invasion, angiogenesis, metastasis and immune-suppression [14]. In our study, both INOS (M1-polarized macrophage markers) and CD163 (M2-polarized macrophage markers) were correlated with the mitotic index, suggesting the distinct involvement of M1- and M2- macrophages in tumor growth. OS development may thus be accompanied by a switch in the phenotype of infiltrating TAMs, from anti-metastatic M1-macrophages to pro-metastatic M2-macrophages. This hypothesis is in agreement with the *in vivo* work described by Xiao et al. who showed a switch in macrophage subpopulations in a mouse model of human OS from M1-macrophages during the first week of tumor growth, to M2-macrophages after 2-3 weeks [23]. In addition, Pahl et al. demonstrated that human M1-like macrophages can be induced to exert direct anti-tumor activity against OS cells, mediated by TNF-α and IL1-β [24]. In the same manner, Ségaliny et al. demonstrated that OS cells expressed IL-34, increasing the recruitment of M2-polarized macrophages into the tumor tissue, which correlates with tumor vascularization and the metastatic process [25]. Our study provides new evidence of the dynamic of macrophage differentiation in the OS microenvironment.

Regarding the metastatic process, our results showed a significantly higher vascular density in OS Meta+ and a correlation between M2-macrophages and the CD146^+^ vascular network, providing explanations regarding tumor growth and the metastatic process. OS cells themselves induce endothelial cell proliferation and neoangiogenesis 26, 27]. The close relationship between hypoxia, TAMs and vascularization is well-known in other cancer types reviewed in many studies [28–30]. The hypoxic OS microenvironment leads to an increase in chemoattractant agents, including HIF-1α (transcription factor for pro-angiogenic factors such as VEGF) and CXCL12 expressed by myeloid cells [31], and induces macrophage differentiation preferentially toward a M1-macrophage phenotype. In return, TAMs play an important role in the angiogenic switch, releasing more than 30 cytokines and angiogenic factors, such as VEGF, b-FGF, IL-8 and IL-6 [32]. This vascularization facilitates the metastatic process, promoting the migration/circulation of tumor cells, thanks to matrix degradation by matrix metalloproteinases (MMP2, MMP7 and MMP9), which are released by tumor cells and by TAMs as well [7, 33]. However, little is known about this direct correlation between TAMs and vascular density in human OS samples. Ségaliny et al. demonstrated that IL-34 promotes the adhesion of mononuclear phagocytes (CD34^+^/monocytes) to activated endothelial cells under physiological shear stress conditions [25]. Guo et al. showed that hypoxia promoted migration and induced CXCR4 expression via HIF-1α activation in human OS [34].

In our study, INOS infiltration and OPG staining were significantly higher in OS Meta- and there was a positive correlation between M1-polarized macrophages and OPG. OPG still plays a controversial role, with both pro- and anti-tumor activity in the bone OS microenvironment [35]. In the same way, there are some conflicting data about the functional relationship between osteoclasts and tumor cells. Avnet et al. highlighted that the occurrence of osteoclasts in OS biopsies was positively associated with aggressive disease (presence of lung metastases at diagnosis) [36], whereas the loss of osteoclasts contributed to the development of pulmonary metastases for other authors [37]. Endo-Munoz et al. suggested an evolution in the microenvironment during tumor growth: osteoclasts contribute to a niche environment in the early stage of OS, nurturing the growth and expansion of the tumor, whereas in the later stage disease, intra-tumor heterogeneity leads to the acquisition of phenotypes that inhibit osteoclastogenesis, and the destruction of the niche, thus permitting metastasis [11]. All these data underline the importance of distinguishing local tumor growth from the metastatic process. According to previous studies targeting atheromatous plaques and bone formation, the link between macrophages and OPG can be explained by the OPG-induced chemotactism of M1-macrophages, and in turn, by the secretion of OPG by endothelial cells [38–41].

The characterization of the microenvironment makes it possible to explain not only tumor growth and the metastatic process, but also to propose therapeutics. Based on the present study, targeting M2-macrophages and blocking their pro-angiogenic activity appears to be an interesting therapeutic approach for OS. On the contrary, the promotion of M1-macrophages may improve overall survival, as is already the case with the macrophage-activating agent, muramyl tripeptide-phosphatidyl ethanolamine (MTP-PE) [24, 42–45]. Finally, several preclinical studies show the benefit of targeting the bone microenvironment, associated with a better response to chemotherapy and a decrease in tumor growth [46–48]. Our study gives additional arguments for active participation of the immune cells, vascularization, and bone constituents in response to chemotherapy. Regarding overall survival, an older age at diagnosis, non-metastatic tumor, good response to chemotherapy and marked TAM infiltration were associated with a better prognosis. Older age, detectable primary metastases, and tumor necrosis after chemotherapy are currently validated as prognostic factors, as are large tumor size, and location (axial or proximal extremity) [1, 49]. As metastasis decreases the diagnosis, we assessed the impact of the prognostic factor for metastasis on overall survival. We observed the significant prognostic impact of TAM on overall survival in OS (whether it is metastatic or not), confirming the data of Buddingh et al. [22]. These results, plus the fact that INOS infiltration is a predictive factor for a non-metastatic process, underline the key role played by TAMs, and their associated subtypes, in OS pathophysiology and outcome. It has also become necessary to characterize each key player in the bone microenvironment at each step/stage of tumor development in order to understand the dynamic evolution, and to adapt the treatment.

## MATERIALS AND METHODS

### Patient cohort

**Figure 3:**
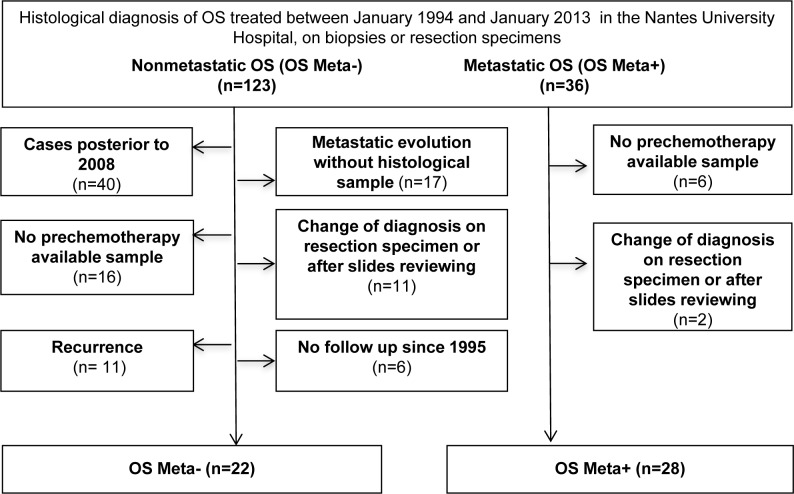
Flow chart of inclusion/exclusion criteria 159 patients were enrolled and after the selective process two groups of patients were defined: 22 non-metastatic patients “OS Meta- group”, and 28 metastatic patients, “OS Meta+ group”.

Patient data were collected from the pathology database at the Nantes University Hospital (France). The experimental procedures were carried out in accordance with both the ethical standards of the responsible institutional committee on human experimentation, and with the Helsinki Declaration (Authorization: French Research Ministry n° 2008-402). All patients with OS treated between January 01, 1994 and January 01, 2013, for whom paraffin-embedded tissue blocks of primary tumors were available, were included. The inclusion and exclusion criteria are summarized in Figure 3. Patients without pre-chemotherapy samples (diagnosis made in another center or in resection specimens) and patients whose diagnosis changed following resection of the specimens or reviewing the slides were excluded. For non-metastatic patients, those with local recurrence, or without follow-up were excluded and a minimum of 5 years' follow-up was required. Two cohorts were finally formed: patients with non-metastatic OS (OS Meta-) and patients with metastatic OS (OS Meta+). For all patients, the following data were collected retrospectively using electronic or paper files, or, if necessary, through direct telephone contact with the general practitioner: demographic data, tumor location, treatment, metastasis (location, synchronous or metachronous) and death (date and cause).

### Tissue microarray preparation and histological analysis

OS tissue samples were formalin-fixed (10%), decalcified and paraffin-embedded. Samples were decalcified with nitric acid or by electrolysis with SAKURA TDE^TM^ 30 (Japan) (98% formic acid, 2% hydrochloric acid). Four µm sections were obtained and stained with hematoxylin eosin saffron (HES). Each primary tumor was classified according to the World Health Organization (WHO) 2013 classification of malignant bone tumors by two independent pathologists [50]. The following histological data were collected: i) location of the primary tumor; ii) in a surgical specimen after neoadjuvant chemotherapy: tumor size, histological response after chemotherapy according to the Huvos score [grades I, II, III and IV characterized by ≤50%, >50% and ≤90%, >90% and ≤99%, and 100% of tumor necrosis respectively, poor responders with a Huvos score of grades I or II, and good responders with grades III or IV] soft tissue invasion (size) [51], quality of resection (R0 or R1), vascular emboli; iii) metastasis: size, quality of resection (R0 or R1). Given how small and precious the tumor samples available were, tissue microarrays (TMAs) were prepared. Three core samples of 1 mm in diameter were performed for each case, in the most representative areas of the HES sections, and then included in paraffin blocks. Three µm sections were stained with HES and used for immunohistochemistry investigations.

### Immunohistochemistry

After systematic optimization of antigen retrieval and antigen detection by primary antibody on human placenta samples decalcified with nitric acid or electrolysis, the following antigens were studied: CD68, pan-macrophages; INOS, M1-macrophage subtype; CD163, M2-macrophage subtype; CD3, CD4 and CD8, T lymphocytes; CD20, B lymphocytes; CD117, mast cells; CD31, CD146, and smooth muscle actin (SMA) for endothelial and perivascular cells; OPG; RANKL and MIB-1 (Ki-67 index) (Supplementary Table 1). CD3, CD4, CD8, CD20, CD68, CD117, SMA, CD31 and Ki-67 antigen were detected using a fully automated immunohistochemical device (DAKO Autostainer Link 48) associated with the Dako EnVision^TM^ Flex detection system. Immunoreactivity was detected by DakoEnVision^TM^ FLEX DAB^+^ Chromogen. The immunoreactivity of CD146, INOS, OPG and RANKL were analyzed using a manual technical process. After deparaffinization and rehydration of the tissue sections, antigen retrieval was carried out at 96°C for 20 h in EDTA or citrate buffer (LabVision^TM^ PT Module, ThermoScientific). Endogenous peroxidase was blocked with hydrogen peroxide followed by blocking the non-specific antibody. Then, the corresponding primary antibody was deposited, antigen positivity was revealed with DAB chromogen and tumor sections were counterstained with hematoxylin. The negative control was analyzed using a similar procedure, excluding the primary antibody and using a normal rabbit-irrelevant IgG (R&D Systems). TMAs slides were scanned and the images were automatically digitized (Nanozoomer, Hamamatsu photonics) before quantification. Immunoreactivity was analyzed qualitatively (cell type, location, nuclear / membrane / cytoplasmic staining) and semi-quantitatively. Semi-quantification was done according to the following criteria: i) for the immune cells: number of cytoplasmic stains on 3 high power (x40) microscopic fields (HPF) in “hot-spots” (areas with high cellular density) per core sample; ii) for vascular, tumor cell density and OPG/RANKL positivity: 0, no staining; 1, < 1/3 of the surface; 2, between 1/3 and 2/3 of the surface; 3, > 2/3 of the surface; iii) for tumor cell proliferation: number of MIB-1 nuclear stains on one HPF. For each case, the mean of the values in the three core samples was performed, and the higher value for vascular or cellular density was selected. For observations and semi-quantifications, a double-blind examination by two experienced pathologists was carried out.

### Statistical analysis

Categorical data were presented as numbers and frequencies and quantitative data were presented with their median and range. To test the link with the presence of metastases, a non-parametric Fisher's test was used for categorical data and a non-parametric Mann-Whitney Wilcoxon's test for quantitative and gradual data. Spearman's correlation coefficient was computed to compare variables in pairs for primary lesions. The Odds ratio was computed to compare quantitative and qualitative variables in pairs for primary lesions. A multivariate logistic regression analysis was used to study the relationship between different variables and the presence of metastases. A Cox model was also used to test the relationship between different variables and patient survival time. Data with missing values were excluded from the statistical analysis. An alpha level of 0.05 was chosen to assess statistical significance. All statistics were performed using R 3.1.0 software.

## SUPPLEMENTARY MATERIAL



## References

[1] The ESMO / European Sarcoma Network Working Group Bone sarcomas: ESMO Clinical Practice Guidelines for diagnosis, treatment and follow-up. Ann Oncol.

[2] Mohseny AB, Hogendoorn PCW (2011). Concise review: mesenchymal tumors: when stem cells go mad. Stem Cells.

[3] Xiao W, Mohseny AB, Hogendoorn PC, Celton-Jansen AM Mesenchymal stem cell transformation and sarcoma genesis. Clin Sarcoma Res.

[4] Messerschmitt PJ, Garcia RM, Abdul-Karim FW, Greenfield EM, Getty PJ Osteosarcoma. J Am Acad Orthop Surg.

[5] Grimaud E, Soubigou L, Couillaud S, Coipeau P, Moreau A, Passuti N, Gouin F, Redini F, Heymann D Receptor activator of nuclear factor kappaB ligand (RANKL)/osteoprotegerin (OPG) ratio is increased in severe osteolysis. Am J Pathol.

[6] Ungefroren H, Sebens S, Seidl D, Lehnert H, Hass R Interaction of tumor cells with the microenvironment. Cell Commun Signal.

[7] Zhu L, McManus MM, Hughes DPM Understanding the Biology of Bone Sarcoma from Early Initiating Events through Late Events in Metastasis and Disease Progression. Front Oncol.

[8] Meads MB, Hazlehurst LA, Dalton WS The bone marrow microenvironment as a tumor sanctuary and contributor to drug resistance. Clin Cancer Res.

[9] Olechnowicz SWZ, Edwards CM Contributions of the host microenvironment to cancer-induced bone disease. Cancer Res.

[10] Quail DF, Joyce JA Microenvironmental regulation of tumor progression and metastasis. Nat Med.

[11] Endo-Munoz L, Evdokiou A, Saunders NA The role of osteoclasts and tumor-associated macrophages in osteosarcoma metastasis. Biochim Biophys Acta.

[12] Theoleyre S, Mori K, Cherrier B, Passuti N, Gouin F, Rédini F, Heymann D Phenotypic and functional analysis of lymphocytes infiltrating osteolytic tumors: use as a possible therapeutic approach of osteosarcoma. BMC Cancer.

[13] Gordon S, Martinez FO Alternative activation of macrophages: mechanism and functions. Immunity.

[14] Lewis CE, Pollard JW Distinct role of macrophages in different tumor microenvironments. Cancer Res.

[15] Qian BZ, Pollard JW Macrophage diversity enhances tumor progression and metastasis. Cell.

[16] Rogers TL, Holen I Tumor macrophages as potential targets of bisphosphonates. J Transl Med.

[17] Alfranca A, Martinez-Cruzado L, Tornin J, Abarrategi A, Amaral T, de Alava E, Menendez P, Garcia-Castro J, Rodriguez R Bone microenvironment signals in osteosarcoma development. Cell Mol Life Sci.

[18] Ando K, Heymann MF, Stresing V, Mori K, Rédini F, Heymann D (2013). Current therapeutic strategies and novel approaches in osteosarcoma. Cancers.

[19] Baud'huin M, Lamoureux F, Duplomb L, Rédini F, Heymann D RANKL, RANK, osteoprotegerin: key partners of osteoimmunology and vascular diseases. Cell Mol Life Sci.

[20] Fidler IJ The pathogenesis of cancer metastasis: the ‘seed and soil’ hypothesis revisited. Nat Rev Cancer.

[21] Paget S The distribution of secondary growths in cancer of the breast. 1889. Cancer Metastasis Rev.

[22] Buddingh EP, Kuijjer ML, Duim RA, Bürger H, Agelopoulos K, Myklebost O, Serra M, Mertens F, Hogendoorn PC, Lankester AC, Cleton-Jansen AM Tumor-infiltrating macrophages are associated with metastasis suppression in high-grade osteosarcoma: a rationale for treatment with macrophage activating agents. Clin Cancer Res.

[23] Xiao Q, Zhang X, Wu Y, Yang Y Inhibition of macrophage polarization prohibits growth of human osteosarcoma. Tumor Biol.

[24] Pahl JHW, Kwappenberg KM, Varypataki EM, Santos SJ, Kuijjer ML, Mohamed S, Wijnen JT, van Tol MJ, Cleton-Jansen AM, Egeler RM, Jiskoot W, Lankester AC, Schilham MW Macrophages inhibit human osteosarcoma cell growth after activation with the bacterial cell wall derivative liposomal muramyl tripeptide in combination with interferon-γ. J Exp Clin Cancer Res.

[25] Ségaliny AI, Mohamadi A, Dizier B, Lokajczyk A, Brion R, Lanel R, Amiaud J, Charrier C, Boisson-Vidal C, Heymann D Interleukin-34 promotes tumor progression and metastatic process in osteosarcoma through induction of angiogenesis and macrophage recruitment. Int J Cancer.

[26] de Nigris F, Mancini FP, Schiano C, Infante T, Zullo A, Minucci PB, Al-Omran M, Giordano A, Napoli C Osteosarcoma cells induce endothelial cell proliferation during neo-angiogenesis. J Cell Physiol.

[27] Schiano C, Grimaldi V, Casamassimi A, Infante T, Esposito A, Giovane A, Napoli C Different expression of CD146 in human normal and osteosarcoma cell lines. Med Oncol.

[28] Dirkx AE, Oude Egbrink MG, Wagstaff J, Griffioen AW (2006). Monocyte/macrophage infiltration in tumors: modulators of angiogenesis. J Leukoc Biol.

[29] Lin EY, Pollard JW Tumor-associated macrophages press the angiogenic switch in breast cancer. Cancer Res.

[30] Lin EY, Li JF, Gnatovskiy L, Deng Y, Zhu L, Grzesik DA, Qian H, Xue XN, Pollard JW Macrophages regulate the angiogenic switch in a mouse model of breast cancer. Cancer Res.

[31] Murdoch C, Muthana M, Coffelt SB, Lewis CE The role of myeloid cells in the promotion of tumor angiogenesis. Nat Rev Cancer.

[32] Guo C, Buranych A, Sarkar D, Fisher PB, Wang XY The role of tumor-associated macrophages in tumor vascularization. Vasc Cell.

[33] Ando K, Mori K, Verrecchia F, Baud'huin Rédini F, Heymann D (2012). Molecular alterations associated with osteosarcoma development. Sarcoma.

[34] Guo M, Cai C, Zhao G, Qiu X, Zhao H, Ma Q, Tian L, Li X, Hu Y, Liao B, Ma B, Fan Q Hypoxia promotes migration and induces CXCR4 expression via HIF-1α activation in human osteosarcoma. PloS One.

[35] Lamoureux F, Moriceau G, Picarda G, Rousseau J, Trichet V, Rédini F Regulation of osteoprotegerin pro- or anti-tumoral activity by bone tumor microenvironment. Biochim Biophys Acta.

[36] Avnet S, Longhi A, Salerno M, Halleen JM, Perut F, Granchi D, Ferrari S, Bertoni F, Giunti A, Baldini N Increased osteoclast activity is associated with aggressiveness of osteosarcoma. Int J Oncol.

[37] Endo-Munoz L, Cumming A, Rickwood D, Wilson D, Cueva C, Ng C, Strutton G, Cassady AI, Evdokiou A, Sommerville S, Dickinson I, Guminski A, Saunders NA Loss of osteoclasts contributes to development of osteosarcoma pulmonary metastases. Cancer Res.

[38] Guihard P, Danger Y, Brounais B, David E, Brion R, Delecrin J, Richards CD, Chevalier S, Rédini F, Heymann D, Gascan H, Blanchard F Induction of osteogenesis in mesenchymal stem cells by activated monocytes/macrophages depends on oncostatin M signaling. Stem Cells.

[39] Heymann MF, Herisson F, Davaine JM, Charrier C, Battaglia S, Passuti N, Lambert G, Gouëffic Y, Heymann D Role of the OPG/RANK/RANKL triad in calcifications of the atheromatous plaques: comparison between carotid and femoral beds. Cytokine.

[40] Mosheimer BA, Kaneider NC, Feistritzer C Expression and function of RANK in human monocyte chemotaxis. Arthritis Rheum.

[41] Mosheimer BA, Kaneider NC, Feistritzer C, Djanani AM, Sturn DH, Patsch JR, Wiedermann CJ Syndecan-1 is involved in osteoprotegerin-induced chemotaxis in human peripheral blood monocytes. J Clin Endocrinol Metab.

[42] Ando K, Mori K, Corradini N, Redini F, Heymann D Mifamurtide for the treatment of nonmetastatic osteosarcoma. Expert Opin Pharmacother.

[43] Chou AJ, Kleinerman ES, Krailo MD, Chen Z, Betcher DL, Healey JH, Conrad EU, Nieder ML, Weiner MA, Wells RJ, Womer RB, Meyers PA, Children's Oncology Group Addition of muramyl tripeptide to chemotherapy for patients with newly diagnosed metastatic osteosarcoma: a report from the Children's Oncology Group. Cancer.

[44] Meyers PA, Chou AJ Muramyl tripeptide-phosphatidyl ethanolamine encapsulated in liposomes (L-MTP-PE) in the treatment of osteosarcoma. Adv Exp Med Biol.

[45] Cathomas R, Rothermundt C, Bode B, Fuchs B, von Moos R, Schwitter M RANK ligand blockade with denosumab in combination with sorafenib in chemorefractory osteosarcoma: a possible step forward?. Oncology.

[46] Lamoureux F, Richard P, Wittrant Y, Battaglia S, Pilet P, Trichet V, Blanchard F, Gouin F, Pitard B, Heymann D, Redini F Therapeutic relevance of osteoprotegerin gene therapy in osteosarcoma: blockade of the vicious cycle between tumor cell proliferation and bone resorption. Cancer Res.

[47] Rousseau J, Escriou V, Lamoureux F, Brion R, Chesneau J, Battaglia S, Amiaud J, Scherman D, Heymann D, Rédini F, Trichet V Formulated siRNAs targeting Rankl prevent osteolysis and enhance chemotherapeutic response in osteosarcoma models. J Bone Miner Res.

[48] Heymann MF, Brown H, Heymann D Drugs in early clinical development for the treatment of osteosarcoma. Expert Opin Investig Drugs.

[49] Burningham Z, Hashibe M, Spector L, Schiffman JD The epidemiology of sarcoma. Clin Sarcoma Res.

[50] (2013). WHO classification of tumors of soft tissue and bone.

[51] Rosen G Preoperative (neoadjuvant) chemotherapy for osteogenic sarcoma: a ten year experience. Orthopedics.

