# Supplementary material for: Dysregulation of macrophage polarization is associated with the metastatic process in osteosarcoma
Source: Oncotarget. 2016 Nov 13;7(48):78343–54. doi: 10.18632/oncotarget.13055 (PMC5346643; doi:10.18632/oncotarget.13055)
Supplement: Supplementary file 1 [file oncotarget-07-78343-s001.pdf]

Dysregulation of macrophage polarization is associated with the metastatic process in osteosarcoma

Supplementary Material

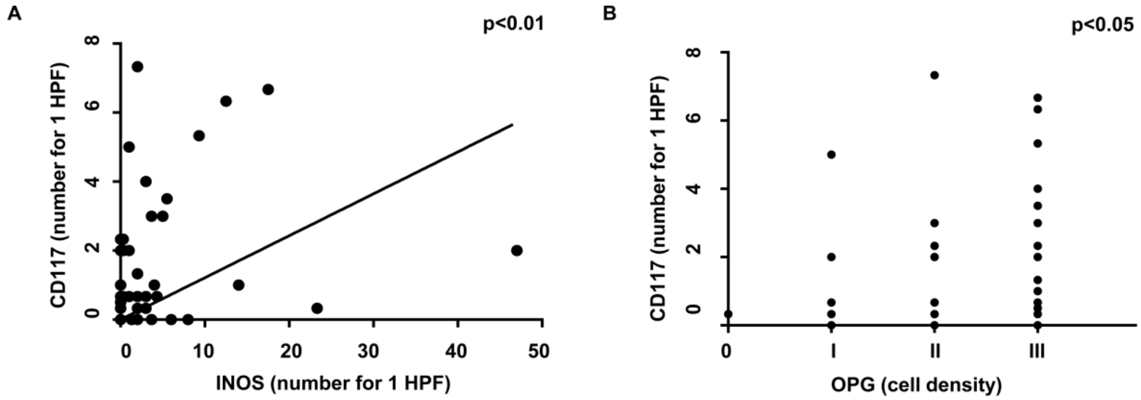

Supplementary Figure 1: Correlation between CD117 expression and INOS (A) or OPG (B) expression in osteosarcoma samples

Supplementary Table 1: Primary antibodies used for the immunohistochemistry study

Human antigen	Antibody (host)	Clone	Source	Dilution
**CD3**	Polyclonal (rabbit)	A052	DAKO	1/100
**CD20**	Monoclonal (mouse)	M0755, L26	DAKO	1/250
**CD4**	Monoclonal (mouse)	NCL- L-CD4-368, 4B12	Novocastra	1/50
**CD8**	Monoclonal (mouse)	DM7103, C8/144B	DAKO	1/50
**CD68**	Monoclonal (mouse)	M0876, PG-M1	DAKO	1/200
**INOS**	Polyclonal (rabbit)	ab15323	Abcam	1/100
**CD163**	Monoclonal (mouse)	NCL-L-CD16, 10D6	Novocastra	1/100
**CD117**	Polyclonal (rabbit)	A4502, c-kit	DAKO	1/750
**CD31**	Monoclonal (mouse)	M082, JC70A	DAKO	1/50
**SMA**	Monoclonal (mouse)	F/MS-113, 1A4	MM France	1/400
**CD146**	Monoclonal (rabbit)	ab75769	Abcam	1/200
**Ki-67**	Monoclonal (mouse)	MIB-1	Dako	1/100
**OPG**	Polyclonal (rabbit)	ab9986	Abcam	1/50
**RANKL**	Polyclonal (goat)	AF626	R&D Systems	1/20

Supplementary Table 2: multivariate logistic regression analysis to study the relationship between different variables and the presence of metastases.

	**Estimate**	**2.5 %**	**97.5 %**	**OR**	**IC 2.5%**	**IC 97.5%**	**Std. Error**	**z value**	**Pr(>|z|)**
Male	2,517	0,0523	6,284256	12,39137	1,054394	536,0651	1,4917	1,687	0,0915
Age	0,08972	0,0112	0,210767	1,093868	1,011872	1,234625	0,0476	1,885	0,0594
Cd68	-0,19883	-0,512	0,022245	0,819689	0,599363	1,022495	0,12761	-1,558	0,1192
cd20	1,29536	-0,441	3,445052	3,652311	0,643191	31,34491	0,92546	1,4	0,1616
**INOS**	-0,80466	-1,732	-0,22411	0,44724	0,176863	0,799228	0,3704	-2,172	**0,0298**
CD117	0,44663	-0,172	1,207732	1,563036	0,84192	3,345886	0,32694	1,366	0,1719
**OPG**	-1,76788	-3,968	-0,38735	0,170694	0,018904	0,678856	0,84609	-2,089	**0,0367**
CD31 (density score)	1,44963	-0,091	3,256526	4,261537	0,912723	25,95919	0,8197	1,768	0,077
CD146 (density score)	1,55114	-0,458	4,542223	4,716844	0,63228	93,89932	1,25641	1,235	0,217

Supplementary Table 3: Cox model to test the relationship between different variables and survival time of patients

	**Coef**	**Exp(coef)**	**Lower.95**	**Upper.95**	**p**
**Histological response to chemotherapy III**	-1.5133	0.2202	0.07328	0.6617	**0.00702**
**Metastasis**	2.4825	11.9715	2.764	51.85	**0.000903**
**Age**	$5.197^{e}-02$	$1.53^{e}+00$	1.0271	1.0803	**<0.0001**
Sex	$5.364^{e}-01$	$1.710^{e}+00$	0.4807	6.0813	0.40738
CD3	$-2.229^{e}-01$	$8.002^{e}-01$	0.6122	1.0460	0.10287
**CD4**	$1.030^{e}+00$	$2.801^{e}+00$	1.3480	5.8190	**0.00578**
CD8	$3.756^{e}-01$	$1.038^{e}+00$	0.8835	1.2202	0.64840
**CD68**	$-5.021^{e}-02$	$9.510^{e}-01$	0.9052	0.9991	**0.04609**
INOS	$-1.006^{e}-02$	$9.900^{e}-01$	0.9310	1.0527	0.74837
CD163	$-7.902^{e}-05$	$9.999^{e}-01$	0.9055	1.1042	0.99875
CD117	$1.642^{e}-01$	$1.178^{e}+00$	0.7769	1.7875	0.43983
Ki-67	2.976e-01	$1.030^{e}+00$	0.9687	1.0957	0.34375
